# Antimicrobial Potential of Caffeic Acid against* Staphylococcus aureus* Clinical Strains

**DOI:** 10.1155/2018/7413504

**Published:** 2018-07-15

**Authors:** Małgorzata Kępa, Maria Miklasińska-Majdanik, Robert D. Wojtyczka, Danuta Idzik, Konrad Korzeniowski, Joanna Smoleń-Dzirba, Tomasz J. Wąsik

**Affiliations:** Department of Microbiology and Virology, School of Pharmacy with the Division of Laboratory Medicine in Sosnowiec, Medical University of Silesia in Katowice, ul. Jagiellońska 4, 41-200 Sosnowiec, Poland

## Abstract

Phenolic compounds constitute one of the most promising and ubiquitous groups with many biological activities. Synergistic interactions between natural phenolic compounds and antibiotics could offer a desired alternative approach to the therapies against multidrug-resistant bacteria. The objective of the presented study was to assess the antibacterial potential of caffeic acid (CA) alone and in antibiotic-phytochemical combination against* Staphylococcus aureus *reference and clinical strains isolated from infected wounds. The caffeic acid tested in the presented study showed diverse effects on* S. aureus* strains with the minimum inhibitory concentration (MIC) varied from 256 *μ*g/mL to 1024 *μ*g/mL. The supplementation of Mueller-Hinton agar (MHA) with 1/4 MIC of CA resulted in augmented antibacterial effect of erythromycin, clindamycin, and cefoxitin and to the lesser extent of vancomycin. The observed antimicrobial action of CA seemed to be rather strain than antibiotic dependent. Our data support the notion that CA alone exerts antibacterial activity against* S. aureus *clinical strains and has capacity to potentiate antimicrobial effect in combination with antibiotics. The synergy between CA and antibiotics demonstrates its potential as a novel antibacterial tool which could improve the treatment of intractable infections caused by multidrug-resistant strains.

## 1. Introduction

Antimicrobial drug resistance is currently one of the major public health problems worldwide. Infections caused by multidrug-resistant (MDR) strains are hard to treat and often turn out to be fatal, especially among hospitalized patients with diminished immunity [1–3]. Due to the steady rise in the incidence of intractable infections with multidrug-resistant strains, there is an immediate need to search for the alternative antimicrobial therapies and new antibacterial agents. It has been documented that many naturally occurring polyphenolic compounds have the capacity to inhibit bacterial growth and to sensitize MDR strains to the bactericidal or bacteriostatic action of wide range of antibiotics [4].

Skin is the largest human organ which can be colonized with antibiotic resistant bacterial strains and these bacteria may cause infections for which limited therapeutic options are available [5–7]. Among them* Staphylococcus aureus* is one of the most common pathogens in both community and hospital associated superficial and deep skin infections. The widespread emergence of multidrug-resistant staphylococci strains compromises common therapeutic strategies based on the broad-spectrum antibiotics, thus worsening infection control. What is more, such therapies greatly affect skin microbiome and may result in further selection of multidrug-resistant nonstaphylococci bacteria [8–10].

Rapidly growing bacterial resistance to antibiotics dictates the ongoing search for an alternative approach to the treatment of intractable infections [11–20]. Our previous studies have shown that phytochemical compounds such as catechin hydrate and protocatechuic acid ethyl ester demonstrate antimicrobial properties against* Staphylococcus aureus* strains [21, 22]. Caffeic acid, a plant phenylpropanoid pathway secondary metabolite, is classified as a hydroxycinnamic acid containing both phenolic and acrylic functional groups and its derivatives include amides, esters, sugar esters, and glycosides. Caffeic acid can be found in many plant products: coffee beverages, argan oil, oats, wheat, rice, and olive oil [3, 23]. It has been reported that CA showed antimicrobial potential and/or synergistic effects with antibiotics against* S. aureus, S. epidermidis, K. pneumoniae, S. marcescens, P. mirabilis, E. coli, P. aeruginosa, B. cereus, M. luteus, L. monocytogenes, *and* C. albicans* strains [20, 24–27].

The majority of studies on antibacterial action of CA or its derivatives have been focused on the reference bacterial strains. Studies on antibacterial potential of CA against clinical isolates are scarce, either with respect to the CA alone or in CA-antibiotic combination. In the face of the observed steady increase in the incidence of nosocomial skin infections caused by bacteria resistant to broad spectrum of antibiotics the objective of the presented study was to assess* in vitro* antibacterial potential of caffeic acid alone and in antibiotic-phytochemical combination, using a panel of* Staphylococcus aureus *clinical strains isolated from intractable infected wounds.

## 2. Materials and Methods

### 2.1. Examined Strains, Media, and Reagents

Twenty* S. aureus *clinical strains were isolated from infected wounds of hospitalized patients, and 3* S. aureus* reference strains:* S. aureus* ATCC 25923,* S. aureus* ATCC 43300, and* S. aureus* ATCC 6538 were obtained directly from ATCC (American Type Culture Collections). To ensure the homogeneity of clinical strains all isolates were derived from the surgical wounds, leading to the relatively small sample size. All examined strains were stored in Tryptic Soy Broth (TSB) medium with 20% of glycerol at -80°C, until further use. Caffeic acid was purchased from Sigma Chemical Co. (St. Louis, MO, USA) and dissolved in DMSO (Sigma Chemical Co.; St. Louis, MO, USA) before use.

### 2.2. Molecular Identification and Characteristic of Clinical Strains

Detection of* dnaJ* gene fragment was performed by restriction fragment length polymorphism (PCR-RFLP). For PCR-RFLP method, bacterial DNA was extracted with the GeneMATRIX Tissue & Bacterial DNA Purification KIT (EURx Ltd., Gdańsk, Poland) according to the manufacturer's recommendations with the modification described by Shah et al. [28]. Briefly, 883 bp region of* dnaJ* gene coding for the N-terminal domain of the receptor was amplified with SA-(F) 5′-GCC AAA AGA GAC TAT TAT GA-3′ and SA-(R) 5′-ATT GTT TAC CTG TTT GTG TAC C-3′ as forward and reverse primers, followed by digestion with 10 U of restriction enzymes* Xap*I and* Bsp*143I (Fermentas, Vilnius, Lithuania) [28]. Digestion patterns were checked against 1 Kb HypeLadderIV (BLIRT SA, Gdańsk, Poland) molecular weight marker and visualized under the UV light.

Detection of* mecA* gene linked to methicillin resistance was done by polymerase chain reaction (PCR) with sequence-specific primers as described previously by Murakami et al. [29]. Briefly, 533 bp coding region of* mecA* gene was amplified with sequence-specific primers: (F) 5′-AAA ATC GAT GGT AAA GGT TGG C-3′ and (R) 5′-AGT TCT GCA GTA CCG GAT TTG C-3′. The PCR amplifications were carried out using 10 × PCR RED master mix kit (BLIRT SA, Gdańsk, Poland) in a MJ Mini Personal Thermal Cycler (Bio-Rad, Hercules, CA, USA). PCR products were electrophoresed in 1.5% agarose gel containing ethidium bromide (Promega, Madison, WI, USA). PCR products size was checked against 1 Kb HypeLadder IV (BLIRT SA, Gdańsk, Poland) molecular weight marker and visualized under the UV light.

### 2.3. Phenotypic Drug Resistance Evaluation

The resistance phenotypes to methicillin were determined according to the disc-diffusion method according to the EUCAST recommendation [30]. Briefly, a bacterial colony suspension equivalent to 0.5 McFarland units was inoculated to Mueller-Hinton agar (MHA, BTL, Łódź, Poland), with a 30 *μ*g cefoxitin disk (EMAPOL, Gdańsk, Poland) and interpreted after 20 h of incubation at 35°C. All strains were classified as MRSA (methicillin-resistant* Staphylococcus aureus*) or MSSA (methicillin-sensitive* Staphylococcus aureus*) by analysis of growth inhibition zone diameter size (<22 and ≥22, respectively) according to the EUCAST recommendation [30].

All examined strains were tested for antimicrobial susceptibility to macrolides and lincosamides by the disk diffusion method according to the EUCAST recommendations [31]. For disk diffusion method, 90 mm plates with the agar medium were inoculated by swabbing the agar with a swab soaked in a bacterial suspension of 1 × 10^8^ cells/mL. The analysis of antimicrobial susceptibility was done with the disks (EMAPOL, Gdańsk, Poland) containing 2 *μ*g clindamycin (DA) and 15 *μ*g erythromycin (E), with distance between disks' edges of 15-16 mm [31]. The growth inhibition zone diameter size was interpreted after 18 h of incubation at 35°C. The isolates were classified as resistant or sensitive based on the zone diameter size and shape.

Vancomycin resistance for all strains was determined with the use of E-test method according to the EUCAST recommendation [32].

### 2.4. Minimum Inhibitory Concentrations Determination with the Microdilution Method

The minimum inhibitory concentrations (MICs) of caffeic acid towards the staphylococcal strains were measured using the standard microdilution liquid method in sterile 96-well polystyrene plates (FL Medical, Torreglia, Italy) in a final volume of 200 *μ*L [33]. The cell concentrations were estimated from the optical densities at 600 nm wavelength with the formula CFU/mL =* A*_600_ (3.8 × 10^8^), where CFU was the number of colony-forming units. One hundred microliters of mid-logarithmic-phase bacterial cultures (5 × 10^5^ CFU/mL) in TSB was added to 100 *μ*L of serially diluted CA (1, 2, 4, 8, 16, 32, 64, 128, 256, and 1024 *μ*g/mL). The stock solution of CA at 4096 *μ*g/l was prepared from CA powder. Serial dilutions were made as follows: 11 wells of 96-well polystyrene plate were filled with TSB, in the next step 100 *μ*l of CA stock solution was added to the first well and mixed thoroughly, subsequently 100 *μ*l was transferred to the next and remaining wells in the same manner, and finally from the last well 100 *μ*l was removed. Wells containing TSB with bacterial inoculum only served as a bacterial growth control (GC). Additional controls included TSB alone, as a medium sterility control, and TSB with different concentrations of CA as a blank. Microplates were incubated at 37°C for 24 h, and the bacterial cell growth was assessed by measuring the optical density of cultures at 600 nm wavelength with a Multiskan EX microplate reader (Thermo Electron Corp., Vantoa, Finland) [34, 35]. The MIC is defined as the lowest compound concentration that yields no visible microorganism growth, and it indicates the resistance of bacteria to an antimicrobial agent and determines the potency of new antimicrobial agents [33]. All experiments were carried out in triplicate.

### 2.5. Combined Effect of CA and Antibiotics on* S. aureus* Strains

All strains were tested for antimicrobial susceptibility according to the EUCAST recommendations by the E-test method, using commercially available MIC Test Strips (Liofilchem, Italy) with antibiotics concentration gradient [32]. For E-test method, 90 mm plates with the MHA were inoculated by swabbing the agar with a swab soaked in a bacterial suspension of 1 × 10^8^ cells/mL. MIC Test Strips containing concentration gradient of erythromycin (E), clindamycin (DA), cefoxitin (FOX), and vancomycin (VA) were used for the analysis of* S. aureus* antimicrobial susceptibility.

The combined effects of CA and antibiotics were evaluated using plates with MHA with a subinhibitory concentration of CA (one-fourth of the MIC of CA) added [36, 37]. The test strips were placed onto the agar surface and gently pressed to ensure contact using the sterile forceps. Plates were incubated at 35°C for 20 h under aerobic condition. After incubation MIC values were read. The susceptibility testing of each antibiotic for all clinical and reference strains was performed in triplicate and the median MIC values were calculated.

In order to assess combined effect of CA and antibiotics, MICs changes were expressed as Δ% and calculated according to the following formula: (MIC of antibiotic - MIC of antibiotic with CA)/MIC of antibiotic x 100%. Obtained Δ% values were presented with the opposite sign (-Δ%) to indicate the reduction or increase of MIC value for antibiotic after addition of CA in comparison with the MIC for antibiotic only.

### 2.6. Statistical Analysis

To compare MICs and MICs changes across MRSA and MSSA U Mann–Whitney test was used, and the Kruskal-Wallis tests were used to compare MICs and MICs changes across strains negative for MLS_B_ (macrolide, lincosamide, and type B streptogramin mechanism), cMLS_B_ (constitutive MLS_B_ mechanism) and iMLS_B_ (inducible MLS_B_ mechanism).

The MIC changes were expressed as a difference between MIC of antibiotic alone and MIC of antibiotic after CA supplementation [Δ= MIC of antibiotic - MIC of antibiotic with CA].

Bonferroni test was used in a* post hoc* analysis. The results of combined effect of CA and antibiotics were submitted to the Wilcoxon Signed-Rank Test. For all tests p ≤ 0.05 was considered as statistically significant. Data were analyzed by use of STATISTICA v. 10.0 software (StatSoft, Polska) on Windows platform (Microsoft Corp., USA).

## 3. Results and Discussion

### 3.1. Identification and Drug Resistance of Examined Strains

All tested isolates were classified as members of* Staphylococcus aureus* species by both classic microbiological and molecular methods. Detection of* mecA* gene and the phenotypic resistance profiles to methicillin, MLS_B_ antibiotics, and vancomycin were performed for all analyzed strains (Table 1).

Fifteen out of 23 (65%) examined staphylococci strains were resistant to methicillin, according to the presence of* mecA* gene, and 11 (48%) demonstrated the constitutive mechanism of resistance to MLS_B_ antibiotics. Nine strains (39%) exhibited both methicillin and MLS_B_ resistance profile, and all strains were sensitive to vancomycin.

### 3.2. Anti-Staphylococcus Action of Caffeic Acid

The antibacterial activity of CA against* S. aureus* strains was observed in all analyzed strains and its magnitude was strain-dependent. The MIC values for CA ranged from 256 to 1024 *μ*g/mL with a median (Me) of 512 *μ*g/mL, lower quartile (LQ) 512 *μ*g/mL, and upper quartile (UQ) 1024 *μ*g/mL (Table 2). The lowest MIC value of 256 *μ*g/mL was detected for* S. aureus* strains number 7 and 20 and all reference strains. CA at concentration of 512 *μ*g/mL inhibited growth of 11 of examined staphylococci, while the concentration of 1024 *μ*g/mL proved to be active against 7 examined strains (Table 2).

There were no significant differences in CA MICs values across MRSA versus MSSA strains (*p* = 0.463), as well as between strains sensitive to MLS_B_ antibiotics and with different phenotypes of MLS_B_ resistance (*p* = 0.949) (Table 2).

### 3.3. Effects of Interaction of Caffeic Acid and Antibacterial Agents against* S. aureus* Strains

Subsequently we examined the effect of CA in the presence of selected antibiotics. Combined* in vitro* interactions of CA and erythromycin (E), clindamycin (DA), cefoxitin (FOX), or vancomycin (VA) are shown in Table 2. A synergistic effect of suppression of examined strains' growth was noted when CA was used in combination with one of three antibiotics: erythromycin (*p* = 0.0004), clindamycin (*p* = 0.0003), and cefoxitin (*p* = 0.0003). The addition of one-fourth of the MIC of CA to the MHA medium generally increased sensitivity of the examined strains to vancomycin, but this effect did not reach the level of statistical significance (*p* = 0.091).

The diminished MICs of E in the presence of CA was observed for 16 of examined strains. Synergistic effect of CA and E was the most visible for* S. aureus* 3, 13, 14, 17, and 20 strains, which under the influence of CA showed high level of sensitivity to E with substantial reduction of MICs by almost 100%. The level of resistance to E was not affected by the CA presence for* S. aureus* ATCC 25923,* S. aureus* ATCC43300, 8, 12, 16, and 19 strains. The increase of E MIC values after CA supplementation was noted for* S. aureus *ATCC 6538 (Table 2).

The most noticeable decreases (near 100%) of DA MICs after CA supplementation were observed for* S. aureus* strains 13, 17, and 20. For seven* S. aureus* strains (*S. aureus *ATCC 43300, 2, 7, 12, 14, 16, and 19) we have not observed DA MICs changes in the presence of CA. Analyzing the other* S. aureus* strains we noted substantial reduction of DA MICs from 27% to 75% (Table 2).

The strongest augmented effect of FOX and CA was noted for* S. aureus* 14, 17, and 20 strains, which showed sensitivity to the lowest concentrations of this antibiotic under the influence of CA with substantial reduction of MICs at almost 100%. For the other examined strains the diminished MICs of FOX after CA supplementation ranged from 25 to 91%. For some strains the CA-FOX combination has not influenced the susceptibility to FOX. The MICs changes after supplementation with CA were not observed in the case of* S. aureus *ATCC 25923, 2, 6, 7, 9, and 12 strains (Table 2).

Analysis of the susceptibility of* S. aureus* strains to VA-CA combination revealed substantial reduction of MICs from 24% to 98% in comparison with MICs of VA alone for 8 staphylococci strains. For* S. aureus *ATCC 43300, 2, and 6 the increase of MICs was noted in the presence of CA. The level of resistance to VA was not affected by the presence of CA for twelve* S. aureus* strains (*S. aureus *ATCC 25923,* S. aureus *ATCC 6538, 3, 4, 8, 9, 10, 12, 15, 16, 18, and 19) (Table 2).

Statistical analysis revealed no significant differences between MICs changes for MRSA versus MSSA strains (E −* p* =0.974; DA −* p* = 0.922; FOX −* p* =0.089; VA −* p* =0.264), as well as for MLS_B_ negative versus cMLS_B_ and iMLS_B_ strains with respect to E −* p* =0.112; DA −* p* =0.943; VA −* p* = 0.368. For cefoxitin, statistical analysis revealed differences among strains with diverse susceptibility to MLS_B_ antibiotics (p = 0.045), but this was not confirmed in the* post hoc* analysis with Bonferroni test (Table 2).

The presented study showed significant synergistic effect of CA in association with E, DA, and FOX. The synergism between CA and VA was also noted, but it did not prove to be statistically significant.

### 3.4. Discussion

The emergence of multidrug-resistant bacterial strains results, in part, from widespread and inappropriate use of broad-spectrum antibiotics for treatment of skin and soft tissue infections. In consequence the steady rise in the incidence of intractable wounds infections in the hospital environment has been widely noticed. Thus the intensive search for new antibacterial compounds and the alternatives to the therapies based on common antibiotics is ongoing. Among many natural sources the polyphenolic plant second metabolites have been explored in this content [38–43]. It has been reported that polyphenols show wide range of promising anti-inflammatory, antimicrobial, antiviral, and antioxidant activities with low toxicity towards human cells [11].

Our study investigated the antibacterial effect of caffeic acid and evaluated whether the supplementation of this natural compound augments the biological action of commonly used antibiotics. Although the antibacterial properties of caffeic acid have been assessed by several authors [3, 9, 11, 14, 25, 44–46], the present study, to the best of our knowledge, is the first to report activity of CA alone and in antibiotic-phytochemical combination against panel of* S. aureus *clinical strains isolated from intractable wounds infections. What is more, contrary to present study where pure caffeic acid has been used, in majority of previous experiments various caffeic acid derivatives have been evaluated [38–43].

Previous studies on the antimicrobial properties of CA against* S. aureus* reference strains in some cases yielded ambiguous results with the different MIC values assessed for the same strains. For example, Fu et al. explored antibacterial activity of CA and 23 caffeic acid amides against* S. aureus *ATCC 6538. The obtained CA MIC values were lower than noted in our study and ranged from 41 to 50 *μ*g/mL indicating that caffeic acid amides had stronger antibacterial activity than pure CA, which suggests that amide groups could enhance the action of CA [3]. Higher value of MIC for caffeic acid (625 *μ*g/mL) was obtained for the same* S. aureus* ATCC 6538 strain by Pinho et al. [11]. In our work the MIC value of* S. aureus* ATCC 6538 (256 *μ*g/mL, Table 2) was one of the smallest compared to the other analyzed strains. However, this strain was relatively insensitive to the synergistic effects of CA and antibiotics (Table 2). The inconsistency in the CA MIC values between authors could be due to the differences in the experiment setup.

The antibacterial effect of CA against other reference* S. aureus* strains: ATCC 29213 and ATCC 25923 was assessed by Vaquero et al. [25] who observed antibacterial activity of CA against* S. aureus *ATCC 29213, while* S. aureus* ATCC 25293 turned out to be resistant to this compound [25]. Interestingly, in our work* S. aureus* ATCC 25293 was one of the most susceptible strains to CA with MIC value of 256 *μ*g/mL. However, it must be noted that Vaquero in his research used the disc-diffusion method to evaluate the sensitivity of the examined strains. In turn Luis et al. examined antibacterial activity of CA against* S. aureus* ATCC 25923 and two clinical MRSA isolates [9]. In their study all examined strains were sensitive to CA (MIC < 250 *μ*g/mL). In our work MIC values for clinical strains ranged from 256 *μ*g/mL to 1024 *μ*g/mL indicating higher resistance to CA. Since clinical strains in our work have been isolated from the intractable infections, it is possible that they were less susceptible to wide range of antimicrobials than these examined by Luis and coauthors. In our study* S. aureus* ATCC 43300 was sensitive to CA action (MIC = 256 *μ*g/mL) (Table 2), but in the experiment presented by Kyaw et al. [14] CA MIC value against this strain was sixteen times higher than in our study, while MIC values for other tested MRSA strains ranged from 512 *μ*g/mL to 1024 *μ*g/mL which is in accordance with data obtained in our study.

Lima et al. examined antibacterial activity of CA alone and in combination with selected antibiotics against* S. aureus* clinical strain isolated from rectal swab. The MIC value obtained for CA in their study was ≥1024 *μ*g/mL. From the wide spectrum of antibiotics which they examined only norfloxacin exhibited synergistic effect with CA, while combined effect of CA and cefoxitin, clindamycin, or erythromycin was not observed [44].

According to Luis et al. the molecular mechanism of CA antimicrobial action is associated with polyphenol-membrane interaction. Using flow cytometry authors showed increased membrane permeability, depolarization of cell membrane, and reduction of respiratory activity in* S. aureus* ATCC 25923 strain in the presence of CA. The authors suggested that CA mechanism of action may be associated with damage of cell membrane integrity and interferes with aerobic metabolism of* S. aureus* cells [9]. Similar conclusions about CA antibacterial activity were drawn by Nguefack et al. and Hayouni et al. [45, 46]. Furthermore, CA as a phenolic acid shows relatively strong nucleophilic properties [25]; thus it can donate an electron pair to electrophile functional groups of plasma membrane proteins and/or lipids, probably leading to membrane function impairment, which is in accordance with the flow cytometry data [45, 46]. This notion is also supported by the observation that CA inhibited *α*-hemolysin secretion of in* S. aureus*, the process which is membrane dependent [25].

It has been shown that among many polyphenolic compounds caffeic acid could be considered as one of the most potent and promising antimicrobial agents. Vaquero et al. noted that CA possesses stronger antibacterial activity than other examined polyphenols: gallic acid, vanillic acid, and protocatechuic acid [25]. Stojković et al. examined CA activity as a food preservative against* S. aureus* contamination. The authors concluded that CA had better antibacterial activity compared to other tested compounds:* p*-coumaric acid and rutin. According to the authors, the higher antimicrobial effect observed for CA could be associated with one more hydroxyl group substituted at the CA phenol ring [23]. Based on our previous studies we can compare antibacterial and synergistic effects of CA to other natural compounds: protocatechuic acid ethyl ester (EDHB) and catechin hydrate (CH) [21, 22]. We demonstrated that CA exhibits stronger antibacterial action against staphylococcal strains than EDHB and CH. What is more, CA exhibited greater synergistic effect with antibiotics than other compounds [21, 22]. Caffeic acid as a hydroxycinnamic acid has propenoic side chain, which makes it much less polar than the protocatechuic acid which is hydroxybenzoic acid. What is more, caffeic acid as less polar compound also exhibits lipophilicity which may contribute to interfering with the permeability of the cell membrane. Andrade et al. in their work proved that *α*-tocopherol as lipophilic compound may damage the phospholipid and protein membrane components essential for its integrity which consequently leads to increased membrane permeability [47]. The experiments with catechins have also proven that antibacterial properties of these compounds are increasing with the number of carbons in the alkyl chain. This CA property can thus facilitate its transport across the cell membrane, which in turn might be related to the stronger antibacterial action [9, 45, 46]. Therefore, the differences in the antibacterial action of CA, CH, and EDHB observed in our works can be explained by the mechanisms described above [21, 25].

Unfortunately, the magnitude of CA antibacterial properties are not species dependent but as our data show differ among* S. aureus* strains. It is highly probable that the diverse sensitivity of the tested staphylococci strains to CA was due to the ontogenetic diversity within the species. The statistical analysis excluded that the differences between MIC values were affected by methicillin resistance profile or phenotype of resistance to MLS_B_ antibiotics. The CA susceptibility does not seem to be influenced by the presence of the* mecA* gene. In the presented experimental setup the influence of the other resistance genes on the observed results' dispersion could not be excluded. However, since CA molecular mechanism is not fully understood yet, we can only speculate on direct or indirect factors engaged in observed differentiation. That strongly points to the necessity for future research on CA-bacterial cell interactions.

Our study on antibacterial potential of caffeic acid showed that CA augments antimicrobial effect of common antibiotics. We showed that CA diminishes MIC values for erythromycin, clindamycin, and cefoxitin in the CA-antibiotic combination (Table 2), while the synergism between CA and vancomycin, though noted, did not reach the level of statistical significance (Table 2). The resistance to MLS_B_ antibiotics did not affect MICs changes after CA addition and the presence of* mecA *gene was also irrelevant.

As in the case of studies on combined effects of CA and antibiotics, which are limited to one work of Lima et al. [44], the number of reports addressing the combined effects of other natural compounds, such as ethanol extract of propolis and selected antibiotics [15], baicalein and ciprofloxacin [16], flavones and *β*-lactam antibiotics [48], berberine and azithromycin, ampicillin, levofloxacin, or cefazolin [49] towards* S. aureus* strains, are relatively small. Published data point out to a promising effect of such phenol-antibiotic combination on clinical staphylococci strains. In the previous studies we have analyzed antibacterial and synergistic effects of other natural compounds: protocatechuic acid ethyl ester (EDHB) and catechin hydrate (CH) [21, 22]. Our results support the notion that CA is one of the most active plant second metabolites and show stronger antibacterial action against staphylococcal strains than EDHB and CH. What is more, CA exhibited greater synergistic effect with antibiotics than other analyzed compounds. Our previous study on antibacterial and synergistic activity of protocatechuic acid ethyl ester on* S. aureus *strains proved significant synergistic effects between EDHB and DA only. The synergism between EDHB-E and EDHB-VA combinations was also noted, but it did not reach the level of statistical significance, while for FOX and EDHB the opposite trend was observed [22]. In case of CH the most noticeable synergistic effect was noted in combination with E and DA. The synergism between CH-VA and CH-FOX combinations was also observed, without reaching the level of statistical significance [21]. On the other hand, the influence of caffeic acid on the antibacterial effect of the above antibiotics showed a substantial reduction of the MICs for three of them (E, DA, and FOX). The synergism between CA and VA was also noted, but it did not prove to be statistically significant.

Our study has some limitations. The number of* S. aureus* strains was relatively small and additional antimicrobials in combination with caffeic acid could be evaluated. However this work was planned as a pilot screening aimed to assess antibacterial potential of caffeic acid against staphylococci clinical strains.

As we mentioned earlier, there is a strong and still growing necessity to find an alternative to the standard antimicrobial therapies. Infections caused by multidrug-resistant strains pose serious and rapidly growing medical problem and so far no satisfying solution has been found [50]. New antibacterial drug discovery and implementation are an extended-in-time and very expensive process. It seems that we have approached the solid wall in finding new classes of antibiotics and/or their chemical derivatives we can base the new therapies on. In this light the plant secondary metabolites could offer a promising alternative [51, 52]. We and the above-mentioned studies showed that natural compounds have lesser antibacterial potential than common antibiotics. The relatively high CA MICs against* S. aureus* clinical strains exclude its application as a single agent to treat infections, but, on the other hand, subinhibitory CA concentration effectively augmented antibacterial activity of common antibiotics allowing for its use in polyphenol-antibiotic combination. Such synergistic effect of CA and antibiotics is especially desired in treatment of intractable wound infections and possibly may contribute to the reduction of the antibiotics side effects.

## 4. Conclusions

Our study indicates that* S. aureus* susceptibility to CA alone, or in the phenolic acid-antibiotic combination, is strain-dependent and is not associated with MRSA and MLS_B_ resistance. The combination of caffeic acid with common antibiotic shows promising synergistic activity against* S. aureus* strains isolated from intractable wound infections which implies the necessity for further research focused on the mechanisms of antimicrobial action of antibiotic CA interactions. Such a research would contribute to the development of the new therapies effective against MDR* S. aureus* clinical strains.

## Figures and Tables

**Table 1:** Antibiotic resistance profiles for tested *Staphylococcus aureus* strains.

Strain	Cefoxitin Diameter of the Inhibition Zone [mm]	Methicillin resistance according to *mecA* presence	Erythromycin Diameter of the Inhibition Zone [mm]	Clindamycin Diameter of the Inhibition Zone [mm]	Mechanism of Resistance to MLS_B_ Antibiotics	The MIC of Vancomycin [*μ*g/mL]	Vancomycin resistance profile
*S. aureus* ATCC 25923	35	MSSA^b^	25	25	-	0.75	S^f^
*S. aureus* ATCC 43300	21	MRSA^c^	0	0	cMLS_B_^d^	0.38	S
*S. aureus* ATCC 6538	31	MRSA	30	30	-	0.50	S
*S. aureus *1^*a*^	34	MSSA	25	25	-	0.75	S
*S. aureus* 2	32	MSSA	23	25	-	0.38	S
*S. aureus* 3	31	MSSA	0	25	iMLS_B_^e^	0.50	S
*S. aureus* 4	32	MRSA	25	27	-	0.50	S
*S. aureus* 5	13	MRSA	0	30	iMLS_B_	0.75	S
*S. aureus* 6	31	MSSA	30	35	-	0.38	S
*S. aureus* 7	32	MRSA	35	33	-	0.50	S
*S. aureus* 8	31	MSSA	30	35	-	0.38	S
*S. aureus* 9	30	MRSA	35	25	-	0.38	S
*S. aureus* 10	31	MSSA	10	22	iMLS_B_	0.38	S
*S. aureus* 11	31	MSSA	21	22	-	0.38	S
*S. aureus* 12	8	MRSA	0	0	cMLS_B_	0.75	S
*S. aureus* 13	14	MRSA	0	0	cMLS_B_	0.75	S
*S. aureus* 14	0	MRSA	0	0	cMLS_B_	0.75	S
*S. aureus* 15	21	MRSA	25	30	-	0.38	S
*S. aureus* 16	18	MRSA	0	0	cMLS_B_	0.50	S
*S. aureus* 17	11	MRSA	0	0	cMLS_B_	0.38	S
*S. aureus* 18	19	MRSA	25	30	-	0.50	S
*S. aureus* 19	14	MRSA	0	0	cMLS_B_	0.50	S
*S. aureus* 20	19	MRSA	0	0	cMLS_B_	0.38	S

^*a*^ *Staphylococcus aureus* 1 ÷ 20: strains from intractable surgical wounds.

^b^MSSA: methicillin-susceptible *S. aureus*.

^c^MRSA: methicillin-resistant *S. aureus*.

^d^cMLS_B_: constitutive macrolide, lincosamide, and type B streptogramin mechanism.

^e^iMLS_B_: inducible macrolide, lincosamide, and type B streptogramin mechanism.

^f^S: sensitive.

**Table 2:** Antibacterial potential of caffeic acid (CA) alone and in antibiotic-phytochemical combination against *Staphylococcus aureus. *The combined effect of CA and antibiotics was evaluated using plates with MHA enriched with the subinhibitory concentration of CA.

Strain^*a*^	CA^b^ MIC^c^ (*µ*g/mL)	E^d^	E+CA	-Δ%^e^	DA^f^	DA+CA	-Δ%	FOX^g^	FOX+CA	-Δ%	VA^h^	VA+CA	-Δ%
*S. aureus* ATCC 25923	256	0.38	0.38	0	0.064	0.047	-27	1	1	0	0.75	0.75	0
*S. aureus* ATCC 43300	256	256	256	0	256	256	0	12	8	-33	0.38	0.50	-32
*S. aureus *ATCC 6538	256	0.064	0.125	95	0.023	<0.016	-30	2	1	-50	0.50	0.50	0
*S. aureus *1	1024	0.50	0.25	-50	0.064	0.032	-50	2	1.5	-25	0.75	0.50	-33
*S. aureus* 2	512	0.50	0.38	-24	0.064	0.064	0	0.75	0.75	0	0.38	0.75	97
*S. aureus* 3	512	256	0.19	<-99.99	0.023	<0.016	-30	1.5	0.38	-75	0.50	0.50	0
*S. aureus* 4	512	0.38	0.19	-50	0.064	0.047	-27	2	1	-50	0.50	0.50	0
*S. aureus* 5	512	256	4	-98	0.094	0.064	-32	256	24	-91	0.75	0.50	-33
*S. aureus* 6	512	0.50	0.125	-75	0.064	<0.016	-75	1.5	1.5	0	0.38	0.50	32
*S. aureus* 7	256	0.38	0.19	-50	0.032	0.032	0	1	1	0	0.50	0.38	-24
*S. aureus* 8	1024	0.19	0.19	0	0.032	<0.016	-50	1.5	0.50	-67	0.38	0.38	0
*S. aureus* 9	512	0.38	0.19	-50	0.064	<0.016	-75	1	1	0	0.38	0.38	0
*S. aureus* 10	512	32	3	-91	0.047	<0.016	-66	2	0.75	-63	0.38	0.38	0
*S. aureus* 11	1024	0.38	0.19	-50	0.047	0.032	-32	1.5	0.75	-50	0.38	0.19	-50
*S. aureus* 12	512	256	256	0	256	256	0	256	256	0	0.75	0.75	0
*S. aureus* 13	512	256	0.125	<-99.99	256	0.094	<-99.99	32	12	-63	0.75	0.50	-33
*S. aureus* 14	1024	256	<0.016	<-99.99	256	<0.016	<-99.99	256	<0.016	<-99.99	0.75	<0.016	-98
*S. aureus* 15	1024	0.25	0.19	-24	0.064	0.023	-64	8	1.5	-81	0.38	0.38	0
*S. aureus* 16	1024	256	256	0	256	256	0	256	32	-88	0.50	0.50	0
*S. aureus* 17	512	256	<0.016	<-99.99	256	<0.016	<-99.99	256	<0.016	<-99.99	0.38	<0.016	-96
*S. aureus* 18	512	0.38	0.094	-75	0.047	<0.016	-66	6	0.75	-88	0.50	0.50	0
*S. aureus* 19	1024	256	256	0	256	256	0	256	64	-75	0.50	0.50	0
*S. aureus* 20	256	256	<0.016	<-99.99	256	<0.016	<-99.99	12	<0.016	<-99.99	0.38	<0.016	-96
Median	512	0.5	0.19	-50	0.064	0.032	-32	2	1	-63	0.50	0.5	0
*p* ^*i*^		*0.0004*			*0.0003*			*0.0003*			*0.091*		

^a^ *Staphylococcus aureus* 1 ÷ 20: strains from intractable surgical wounds.

^b^CA: caffeic acid.

^c^MIC: minimum inhibitory concentration.

^d^E: erythromycin.

^e^MICs changes according to the formula [(MIC of antibiotic - MIC of antibiotic with CA) / MIC of antibiotic] x 100%.

^f^DA: clindamycin.

^g^FOX: cefoxitin.

^h^VA: vancomycin.

^i^Wilcoxon Signed-Rank Test.

## Data Availability

The data supporting the results reported in the presented manuscript are available at Department of Microbiology and Virology, School of Pharmacy with the Division of Laboratory Medicine in Sosnowiec, Medical University of Silesia in Katowice, ul. Jagiellońska 4, 41-200 Sosnowiec, Poland, and under Tomasz J. Wąsik's e-mail: twasik@sum.edu.pl.
